# Descended Social Anxiety Disorder and Craving in Women Heroin Dependence Through Exercise Alerts Plasma Oxytocin Levels

**DOI:** 10.3389/fpsyt.2021.624993

**Published:** 2021-11-26

**Authors:** Jing Song Wang, Jing Lin Liu, Jun Zhang, Jun Tan, Ting Huang, Chun Xia Lu, Xi Yang Peng, Yin Guo, Lan Zheng

**Affiliations:** ^1^Key Laboratory of Physical Fitness and Sports Rehabilitation of the Hunan Province, College of Physical Education, Hunan Normal University, Changsha, China; ^2^Hunan Judicial Police Vocational College, Changsha, China; ^3^College of Physical Education, Hunan International Economics University, Changsha, China

**Keywords:** exercise, oxytocin, SAD, VAS, DDQ, craving

## Abstract

**Purpose:** This study explored the association between peripheral blood oxytocin (OT) and social anxiety disorder (SAD) and cue-induced cravings in female heroin addicts. The effect of exercise on alleviation of SAD and OT levels was also explored.

**Methods:** A total of 72 females with heroin dependence were assigned to three groups based on SAD severity. The three groups were Non-SAD control, SAD control, and SAD exercise groups. Subjects in the SAD exercise group underwent aerobic exercise and resistance training for 8 weeks (60 min/day, 5 days/week). Enzyme-linked immunosorbent assay analysis and Liebowitz Social Anxiety Scale (LSAS) scores were used to determine plasma OT concentration and SAD, respectively. Cue-induced craving was assessed using Visual Analog Scale (VAS) and Desires for Drug Questionnaire (DDQ). Mixed-effect analysis of variance and Pearson correlation analysis were used to explore the effect and correlation between different parameters.

**Results:** OT levels in the SAD exercise group were significantly high after exercise (*p* < 0.01). LSAS, VAS, and DDQ (“Desire and Intention” and “Negative reinforcement”) scores in the SAD exercise group were significantly lower after exercise (*p* < 0.01). Plasma OT level was negatively correlated with LSAS score (*r* = −0.534, *p* < 0.001), VAS score (*r* = −0.609, *p* < 0.001), “Desire and Intention” score (*r* = −0.555, *p* < 0.001), and “Negative reinforcement” score (*r* = −0.332, *p* < 0.01) and positively correlated with the “control” score (*r* = 0.258, *p* < 0.05). LSAS was positively correlated with VAS score (*r* = 0.588, *p* < 0.001) and “Desire and Intention” score (*r* = 0.282, *p* < 0.05).

**Conclusions:** The findings of the present study indicate that plasma OT is a potential peripheral biomarker for prediction of the severity of social anxiety in female heroin withdrawal patients. Aerobic exercise combined with resistance training plus incremental load for 8 weeks can increase plasma OT levels and significantly reduce severity of SAD and cue-induced cravings in female heroin addicts.

## Introduction

Social anxiety disorder (SAD) is mainly caused by a combination of environmental and genetic factors. SAD is characterized by discomfort in various social contexts, such as public speaking, interaction with visitors, or dealing with strangers. Studies report higher incidence of SAD in women relative to men (1–3). People with severe SAD exhibit various physical reactions such as headaches, cold sweats, and gastrointestinal discomfort, which may lead to social avoidance (4). In addition, severe SAD can lead to substance use disorders (SUD) (5). A previous study reported that SAD affects the rate of relapse among drug addicts to some extent. Individuals with SAD have a high risk of developing SUD (6). The study reports that patients with SAD have a two-fold risk of using heroin compared with subjects without SAD (7). SAD is common among patients seeking SUD treatment. A previous study reported that a quarter of patients seeking opiate dependence treatment exhibited SAD (8). In addition, a study reported patients who received pharmacotherapy for opiate dependence had higher SAD levels compared with controls (9). Moreover, findings indicate that half of substance dependence patients have clinically elevated SAD (10). Studies indicate that heroin dependence is associated with social avoidance during withdrawal owing to the high level of physical dependence and emotional disorders (11).

OT is a neuropeptide that is released into the bloodstream from the posterior pituitary and it exhibits several central and peripheral blood effects (12). In addition, OT regulates several aspects of social cognition, social behaviors, and fear conditioning. These effects result in SAD and other disorders associated with impaired social functioning (13). OT levels are high in females relative to the level in males (14). Notably, OT protects females from SAD. Hoge reported low plasma OT levels in patients with a generalized SAD during a pro-social laboratory task paradigm (15). A study by Meyer-Lindenberg indicated that increased levels of OT may prevent SAD (16).

Moreover, OT plays a vital role in establishing clinical experimental models of addiction, relapse, and craving. Studies report that daily peripheral blood OT administration lowers self-administration of heroin in heroin-tolerant rats (17). In addition, OT can attenuate craving and alleviate withdrawal symptoms in female heroin-dependent patients; thus, it is a potential therapy for heroin dependence (18). Furthermore, OT decreases stress and cued reinstatement of opioid seeking (19). OT improves multiple aspects of social functioning, including emotion recognition (20) as well as social abilities in patients with opioid use disorder. These effects ultimately promotes engagement of the patients in psychosocial treatments and social support systems (21).

Regular aerobic exercise increases the plasma OT level in female mice, reduces anxiety, and increases emotions (22). Jazaieri reported that aerobic exercise can improve SAD (23). Effect of exercise on drug addiction and relapse process has been extensively studied and studies report that Qigong can prevent heroin detoxification without side effects (24). In addition, a significant association was observed between exercise and substance abuse in women compared with men (25). SAD and heroin dependence are correlated with OT levels.

The aim of the current study was to explore whether the degree of SAD in heroin withdrawal subjects was correlated with the level of peripheral blood OT. Moreover, the effect of exercise on peripheral blood OT levels, SAD levels, and cue-induced cravings in heroin withdrawal subjects was explored.

## Study Subjects and Methods

### Subjects

A total of 72 subjects with opioid dependence (aged 35–50 years) attending the Female Detoxification Rehabilitation Center of Baimalong in Hunan province were enrolled in this study. Interviews were conducted following guidelines by Structured Clinical Interviews and Statistical Manual (The Diagnostic and Statistical Manual of Mental Disorders-V, DSM-V) to explore whether participants had SAD. Structured interviews were conducted by clinical psychologists. ADIS-IV-Lifetime (ADIS-IV-L) (26) is a structured interview guideline designed to assess current and past (lifetime) diagnoses of anxiety disorders. It allows differential diagnosis of anxiety disorders based on DSM-V criteria. Anxiety Disorders Interview Schedule Lifetime Version (ADIS-IV-L) manual is highly effective for principal diagnosis of SAD (27). Diagnosis of SAD and collection of severity data were carried out using the Liebowitz SAD Scale-self-reported (LSAS-SR) scale. Individuals without SAD were included after screening using the same procedure. SAD patients comprised patients with a score ≥60, whereas Non-SAD included individuals with a score <30 based on the LSAS-SR scale (28, 29).

Exclusion criteria were as follows: (1) history of neurological, psychiatric disease and diagnosis of other mental disorders, except SAD; (2) dependence on other substances; (3) participants diagnosed with bone, muscle, and cardiovascular diseases; (4) individuals with vision and hearing impairment; (5) participants who have taken β-blockers a week before the study; and (6) participants who had not been assessed using Physical Activity Readiness Questionnaire Plus (PAR-Q+).

**Figure 1:**
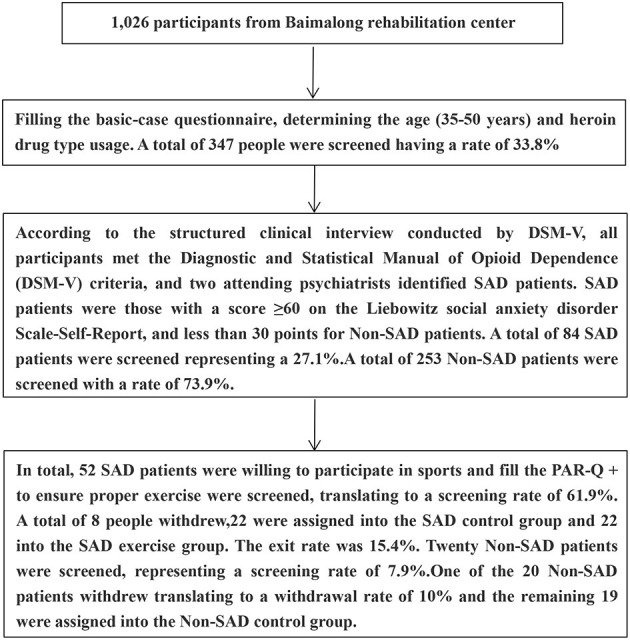
A flowchart of the participant screening process.

This study was conducted in accordance with the Declaration of Helsinki. Approval to conduct the study was obtained from the Institutional Review Committee of Hunan Normal University. A total of 52 SAD patients were randomly assigned into two groups: 26 in the control group and 26 in the exercise group. Eight people withdrew from the study owing to force majeure reasons such as family reasons, blood draw failure, and voluntary abandonment. The withdrawal rate was 15.3%. Twenty-two out of the 44 participants who left after withdrawal were assigned into the SAD control group and 22 participants were assigned into the SAD exercise group. In addition, 1 out of the 20 Non-SAD participants withdrew with a withdrawal rate of 10%; thus, 19 participants were assigned into the Non-SAD control group. See Figure 1 for the participant screening process flowchart. The demographic characteristics of heroin addicts are shown in Table 1.

**Table 1:** Demographic characteristics of heroin addicts.

**Variable**		**Non-SAD control**	**SAD control**	**SAD exercise**
		**(*n* = 19)**	**(*n* = 22)**	**(*n* = 22)**
**Demographic and health characteristics**				
Age (years)		40.26 ± 4.86	38.36 ± 5.69	39.55 ± 5.55
Height (cm)		160.21 ± 4.53	160.82 ± 4.69	159.32 ± 5.15
Weight (kg)		58.93 ± 6.11	59.93 ± 6.81	56.35 ± 5.15
BMI (kg/m^2^)		27.71 ± 5.01	22.19 ± 1.60	23.21 ± 2.90
Waist (cm)		81.89 ± 6.70	82.50 ± 8.38	77.64 ± 4.35
Culture	Primary school or less	4 (21.1)	3 (13.6)	3 (13.6)
	Junior high school	7 (36.8)	9 (40.9)	13 (59.1)
	Senior middle school	5 (26.3)	5 (22.7)	6 (27.2)
	College or above	3 (15.8)	5 (22.7)	0 (0)
Occupation	Unemployed	2 (10.5)	10 (45.5)	15 (68.2)
	Self-employed	4 (21.1)	7 (31.8)	3 (13.6)
	Service	5 (26.3)	2 (9.1)	2 (9.1)
	Manual worker	4 (21.1)	1 (4.5)	1 (4.5)
	General staff	4 (21.1)	2 (9.1)	1 (4.5)
Marital status	Married	10 (52.6)	4 (18.2)	2 (9.1)
	Single	8 (42.1)	15 (68.2)	10 (45.5)
	Widowed	0 (0)	0 (0)	1 (4.5)
	Divorced	1 (5.3)	3 (13.6)	9 (40.9)
Regions	Urban	12 (63.2)	10 (45.5)	10 (45.5)
	Rural	7 (36.8)	12 (54.5)	12 (54.5)
Drug data				
Heroin use at one time (g)		0.52 ± 0.33	0.56 ± 0.33	0.45 ± 0.23
Times per month		20.46 ± 10.42	19.68 ± 11.52	21.25 ± 10.68
Duration (months)		116.41 ± 61.54	116.71 ± 64.35	12.34 ± 71.66
Relapse times		1.15 ± 0.57	1.58 ± 0.71	2.16 ± 1.17

### Procedure

This study was conducted from December 2018 to May 2019. In December 2018, the corresponding author conceptualized the study and contacted the rehabilitation center. Data on basic information and drug use were obtained from female patients attending the Baimalong Female Drug Rehabilitation Center in Hunan Province in January 2019. SAD diagnosis was performed on subjects who met the inclusion criteria, and data on drug use status and medical health conditions were obtained in February 2019. Clinical psychologists made judgments on patient status using structured interviews and SAD questionnaires. Pre-exercise screening and participation willingness surveys were conducted on selected subjects in February 2019 to ensure the suitable exercise is used and to confirm voluntary participation. The exercise intervention was conducted for 8 weeks. Preliminary blood collection, administration of SAD questionnaire, craving questionnaire survey, and exercise intervention were carried out in March 2019. Post-stage blood collection, administration of SAD questionnaire, craving questionnaire survey, and exercise intervention were carried out in May 2019.

### Exercise Intervention

SAD exercise group received exercise intervention whereas subjects in the Non-SAD control and SAD control groups received safety and health education. Exercise intervention comprised aerobic combined resistance training according to ACSM guidelines (30). Each exercise session was conducted for a duration of 8 weeks (60 min/day, 5 days/week). The 60-min exercise program was conducted as follows: warm-up for 5 min, aerobic exercise on a treadmill or spinning exercise for 30 min, weight training for the main muscle groups (arms, chest, back, and legs) for 20 min, and stretching and relaxing for 5 min. A heart rate monitor (PolarTM RS400, Polar Inc, USA) was used to continuously monitor the heart rate throughout the training process. Monitoring of heart rate was conducted to ensure 30 min of continuous aerobic exercise in a predetermined heart rate zone. The treadmill rotation speed in the first 2 weeks was set at a target intensity of 40–60% heart rate reserve (HRR) (31). Subsequently, the treadmill speed was adjusted to a target intensity of 50–70% HRR in the third week. Intensity of weight training was 10–15RM. Four experienced physical exercise coaches directly supervised the training program.

### Physical Activity Readiness Questionnaire Plus

PAR-Q+ is a four-page document comprising questions designed to identify possible restrictions or limitations for physical activity participation. Use of PAR-Q+ helps doctors and sports professionals to explore eligibility of participants before they undergo exercise training.

### Liebowitz Social Anxiety Disorder Scale

LSAS (32) is a 24-item interviewer-rated instrument used for assessment of fear/anxiety and to prevent specific social situations. Respondents were required to rate their fear/anxiety (LSAS-Anxiety subscale) on a four-point scale ranging from 0 (none) to 3 (severe) (first column). Furthermore, participants rated their avoidance level (LSAS-Avoidance subscale) based on a four-point scale ranging from 0 (never) to 3 (usually) (second column). LSAS-SR (self-reported) was used in the present study as it is suitable for Chinese patients and has high reliability and validity. LSAS-SR has good sensitivity and specificity in diagnosis of SAD (33).

### Craving Analysis

#### Desires for Drug Questionnaire

Self-evaluation of instant cravings among female subjects with heroin dependence was performed using DDQ. The questionnaire comprises three dimensions, namely, desire and intention (question numbers 1, 2, 4, 6, 9, 12, and 13), negative reinforcement (question numbers 5, 8, 10, and 11), and the control (question numbers 3 and 7).

#### Visual Analog Scale

VAS is used to visually assess immediate desire for heroin by patients. A 0- to 100-mm VAS scale was adopted for determining the degree of cue-induced craving (0 means “no craving,” 100 means “extreme craving”). The patient was requested to relax for 5 min and then watch neutral pictures and videos for 5 min. Furthermore, the patient was requested to watch pictures and videos of objects, utensils, and heroin inhalation and play sound effects for 5 min. The heart rate and blood pressure were then determined immediately after cue induction was completed. VAS and DDQ were administered immediately after induction process. Psychological scale assessments and blood index testing were conducted a day before conducting the exercise intervention and a day after termination of exercise intervention.

### Enzyme-Linked Immunosorbent Assay

Blood samples were collected from participants between 8:00 and 9:00 a.m. in the morning on the day before and the day after exercise intervention. Subjects who underwent blood sample collection starved from 8:00 p.m. the previous evening. Two milliliters of venous blood was collected into an EDTA anticoagulant test tube. The blood samples were mixed with anticoagulant before centrifugation at 4,000 rpm for 4 min. The supernatant was obtained and stored in an ultralow-temperature refrigerator at −80°C for further analysis. Blood indicators were analyzed at the Shanghai Enzyme Link Company.

### Statistical Analysis

Mixed-effect analysis of variance was performed to explore the effect of group and time before and after exercise on the LSAS, VR-VAS, DDQ scores, and plasma OT levels in heroin-dependent females. Bonferroni method was used in the post-test for cases where *p* < 0.05 for pairwise comparisons between groups. Pearson correlation analysis was utilized to explore the relationship between plasma OT levels and LSAS, VAS, and DDQ scores of the three groups. All statistical analyses were conducted using SPSS 20.0 software. Data were expressed as mean ± standard deviation (M ± SD).

## Results

### Results of LSAS, VR-VAS, and DDQ Pre-exercise and Post-exercise

Mixed-effect analysis of variance was conducted to explore the effects of groups and time on LSAS score. The results showed that group and time significantly affected LSAS score (*p* < 0.001), and a significant interaction was observed between group and time (*p* < 0.001). The LSAS score of the Non-SAD control group was lower relative to the LSAS scores of the SAD control (95% CI: −52.98, −43.72) and SAD exercise groups (95% CI: −54.12, −44.86) (*p* < 0.001) during the pre-exercise period. LSAS scores for the SAD control group and the SAD exercise group were not significantly different during the pre-exercise period (*p* > 0.05). LSAS score for the SAD exercise group was significantly lower compared with the LSAS score for the SAD control group (*p* < 0.001, 95% CI: −30.38, −17.90) during the post-exercise period. Notably, the LSAS score of the SAD exercise group was significantly lower after exercise compared with the LSAS score before the exercise (*p* < 0.001, 95% CI: 22.59, 30.86) (Table 2).

**Table 2:** The effect of exercise on LSAS score.

**Group**	**Pre-exercise**	**Post-exercise**	***p* (intergroup)**	***p* (time)**	***p* (interaction)**
Non-SAD control	22.11 ± 2.87	21.58 ± 3.31	<0.001	<0.001	<0.001
SAD control	70.45 ± 7.44^ΔΔ^	69.00 ± 7.00^ΔΔ^			
SAD exercise	71.59 ± 6.41^ΔΔ^	44.86 ± 12.02^▴▴^^,^^**^			

ΔΔ *p < 0.01, compared with the Non-SAD control group*;

▴▴ *p < 0.01, compared with the SAD control group*;

** *p < 0.01, compared with pre-exercise*.

The effects of group and time on VAS were explored through mixed-effect analysis of variance. The results showed a significant effect of group and time on VAS (*p* < 0.001), and a significant interaction was observed between group and time (*p* < 0.001). VAS score of the Non-SAD control group was significantly lower relative to VAS score of SAD control group (95% CI: −26.74, −7.76) and the VAS score of the SAD exercise group during the pre-exercise period (95% CI: −22.93, −3.95) (*p* < 0.001). However, VAS scores of the SAD control group and the SAD exercise group were not significantly different (*p* > 0.05, 95% CI: −5.32, 12.96). The VAS score in the SAD exercise group was significantly lower compared with degree of craving for drugs in the SAD control group (*p* < 0.001, 95% CI: −21.65, −6.26) post-exercise. The VAS score of the SAD exercise group at 8 weeks was significantly lower compared with the VAS score before the exercise (*p* < 0.001, 95% CI: 7.93, 16.71) (Table 3).

**Table 3:** The effect of exercise on VAS.

**Group**	**Pre-exercise**	**Post-exercise**	***p* (intergroup)**	***p* (time)**	***p* (interaction)**
Non-SAD control	24.47 ± 8.02	24.47 ± 9.31	<0.001	<0.001	<0.001
SAD control	41.72 ± 14.35^ΔΔ^	39.55 ± 11.49^ΔΔ^			
SAD exercise	37.91 ± 13.19 ^ΔΔ^	25.59 ± 9.36^▴▴^^,^^**^			

ΔΔ *p < 0.01, compared with the Non-SAD control group*;

▴▴ *p < 0.01, compared with the SAD control group*;

** *p < 0.01, compared with pre-exercise*.

The results showed no significant difference between the “Desire and Intention” and “Negative reinforcement” “Control” scores of the Non-SAD control group, the SAD control group, and the SAD exercise group before the exercise (*p* > 0.05). Subjects in the SAD exercise group showed significantly lower “Negative reinforcement” scores compared with the scores for the Non-SAD control subjects (*p* < 0.01, 95% CI: −1.66, −0.24) and the SAD control group after the exercise (*p* < 0.05, 95% CI: −1.49, −0.12). Scores for “Desire and Intention” (95% CI: 0.40, 1.23) and “Negative reinforcement” (95% CI: 0.35, 1.29) of the SAD exercise group after the exercise were significantly lower compared with the scores before the exercise (*p* < 0.01; Table 4).

**Table 4:** The effect of exercise on DDQ.

**Dimension**	**Group**	**Pre-exercise**	**Post-exercise**	***p* (intergroup)**	***p* (time)**	***p* (interaction)**
Desire and Intention	Non-SAD control	3.79 ± 0.92	3.95 ± 0.98	0.604	<0.05	<0.01
	SAD control	4.19 ± 1.24	4.07 ± 0.93			
	SAD exercise	4.34 ± 1.02	3.52 ± 0.91^**^			
Negative reinforcement	Non-SAD	4.39 ± 0.96	4.20 ± 1.10	0.063	<0.05	<0.05
	SAD control	4.21 ± 1.09	4.21 ± 0.91			
	SAD exercise	4.23 ± 0.93	3.41 ± 0.79^ΔΔ^^▴^^**^			
Control	Non-SAD control	3.63 ± 1.56	3.71 ± 1.18	0.228	0.276	0.958
	SAD control	2.97 ± 1.49	3.14 ± 1.26			
	SAD exercise	3.02 ± 1.29	3.16 ± 1.15			

ΔΔ *p < 0.01, compared with the Non-SAD control group*;

▴ *p < 0.05, compared with the SAD control group*;

** *p < 0.01, compared with pre-exercise*.

### Pre-exercise and Post-exercise Plasma OT Levels

The effects of group and time on plasma OT levels were explored through mixed-effect analysis of variance. The results showed that group significantly affected the level of plasma OT (*p* < 0.001); however, time point showed no significant effect on plasma OT level (*p* > 0.05). Notably, the findings showed a significant interaction between group and time (*p* < 0.001). The plasma OT level of the Non-SAD control group was higher relative to plasma OT levels of the SAD control group (*p* < 0.01, 95% CI: 1.54, 8.77) and the plasma OT levels of the SAD exercise group before the exercise (*p* < 0.05, 95% CI: 0.32, 7.55). However, the results showed no significant difference in the plasma OT level between the SAD control group and the SAD exercise group before the exercise (*p* > 0.05, 95% CI: −8.59, −3.02). However, the plasma OT level of the SAD exercise group was significantly higher compared with the plasma OT level of the SAD control group after the exercise (*p* < 0.01, 95% CI: 4.48, 12.72). The plasma OT level of the SAD control group was significantly lower relative to that of the Non-SAD control group after exercise (*p* < 0.05, 95% CI: −9.20, −0.24). The plasma OT level of the SAD exercise group showed significant increase after exercise compared with the pre-exercise plasma OT level (*p* < 0.001, 95% CI: −8.59, −3.02) (Table 5).

**Table 5:** The effect of exercise on plasma OT levels (pg/ml).

**Group**	**Pre-exercise**	**Post-exercise**	***p* (intergroup)**	***p* (time)**	***p* (interaction)**
Non-SAD control	60.74 ± 3.91	58.93 ± 3.94	<0.001	0.332	<0.001
SAD control	55.58 ± 5.35^ΔΔ^	54.01 ± 6.40^Δ^			
SAD exercise	56.80 ± 4.62^Δ^	62.61 ± 5.86^▴▴^^,^^**^			

Δ *p < 0.05, compared with the Non-SAD control group*;

ΔΔ *p < 0.01, compared with the Non-SAD control group*;

▴▴ *p < 0.01, compared with the SAD control group*;

** *p < 0.01, compared with pre-exercise*.

### Correlation Analysis Results of Plasma OT Levels and LSAS, VAS, and DDQ Scores

**Figure 2:**
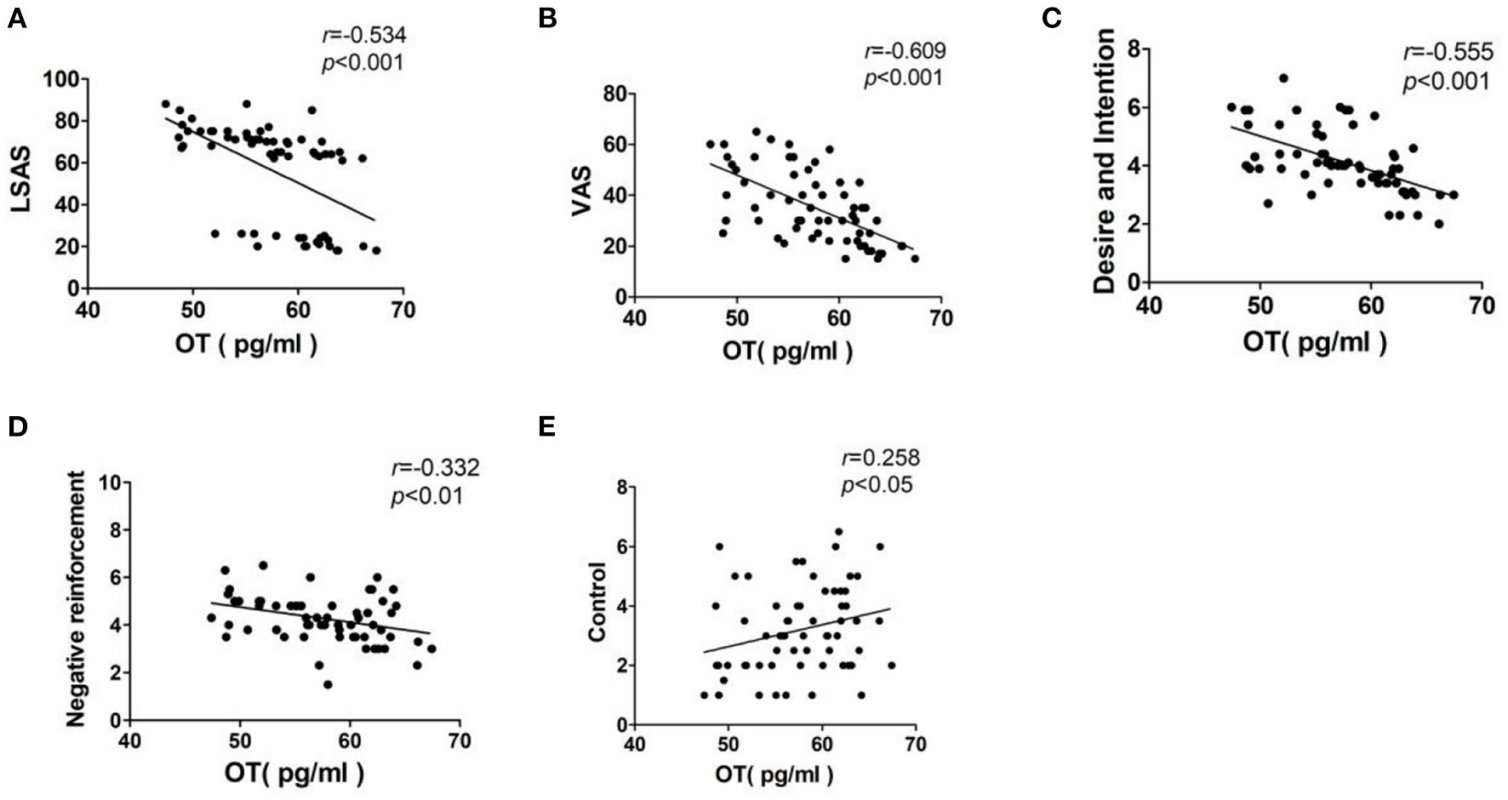
Correlation analysis of plasma OT levels and the LSAS, VAS, and DDQ scores. Interpretation of *r*-value: 0 = no relationship; 0.01–0.19 = no/negligible relationship; 0.20–0.29 = weak positive correlation; 0.30–0.39 = moderate positive correlation; 0.40–0.69 = strong positive correlation; 0.70 or higher = very strong relationship. **(A)** LSAS, Liebowitz social anxiety disorder scale; **(B)** VAS, Visual Analog Scale; **(C)** Desire and Intention, desire for heroin; **(D)** Negative reinforcement, repeated use of heroin to relieve withdrawal symptoms; **(E)** Control, the ability to control heroin.

Pearson correlation analysis was used to analyze the correlation between the plasma OT concentration and the LSAS, VAS, and DDQ scores. The results showed that the plasma OT concentration was negatively correlated with the LSAS score (*r* = −0.534, *p* < 0.001) and VAS score (*r* = −0.609, *p* < 0.001). Besides, plasma OT concentration was negatively correlated with both “Desire and Intention” score (*r* = −0.555, *p* < 0.001) and “Negative reinforcement” (*r* = −0.332, *p* < 0.01). Moreover, there was a positive correlation between the plasma OT concentration and the “Control” score (*r* = 0.258, *p* < 0.05; Figure 2).

**Figure 3:**
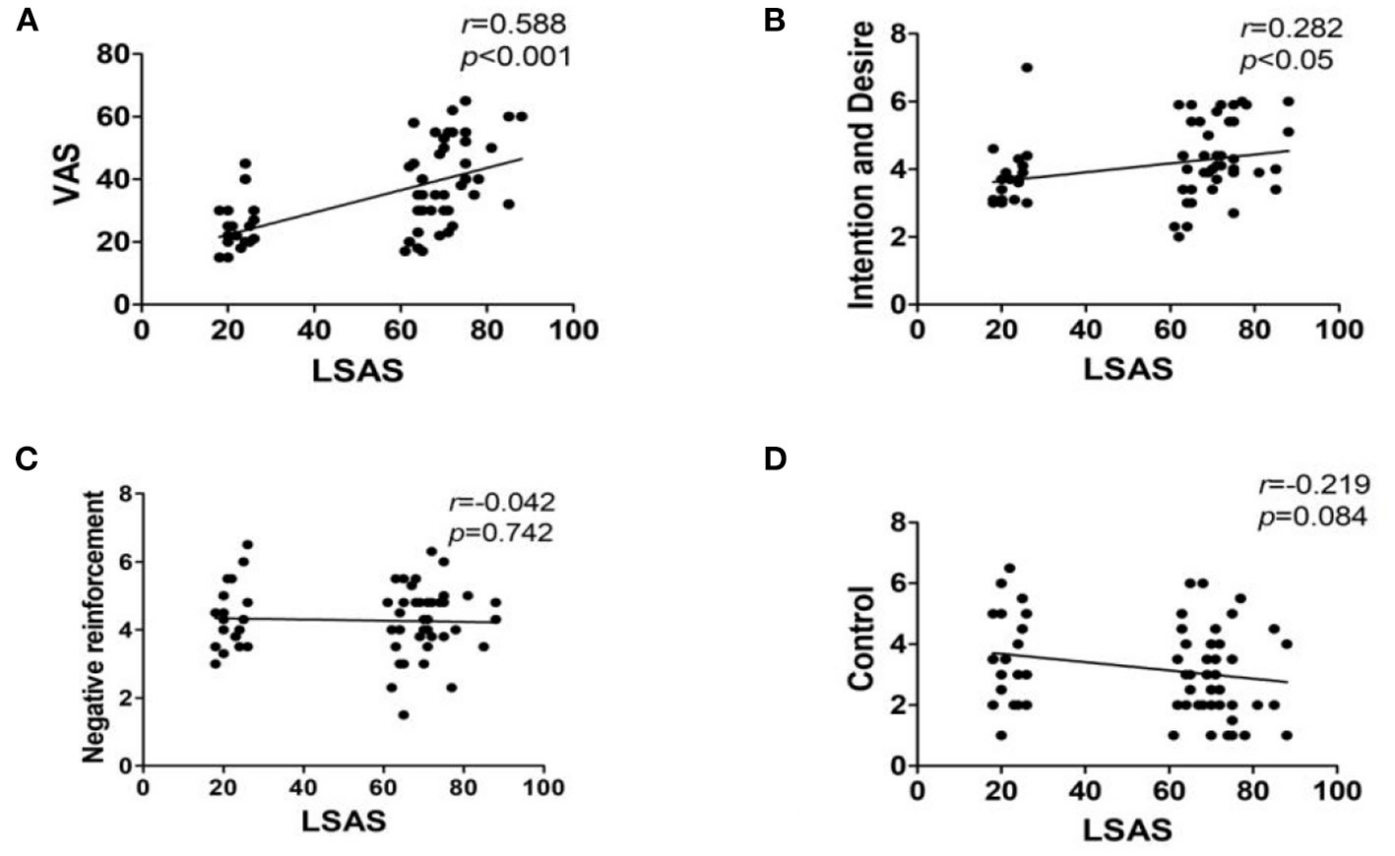
Correlation analysis results of LSAS and the VAS, as well as DDQ scores. Interpretation of *r*-value: 0 = no relationship; 0.01–0.19 = no/negligible relationship; 0.20–0.29 = weak positive correlation; 0.30–0.39 = moderate positive correlation; 0.40–0.69 = strong positive correlation; 0.70 or higher = very strong relationship. **(A)** VAS, Visual Analog Scale; **(B)** Desire and Intention, desire for heroin; **(C)** Negative reinforcement, repeated use of heroin to relieve withdrawal symptoms; **(D)** Control, the ability to control heroin.

Pearson correlation analysis was used to analyze the correlation between the LSAS score and the VAS, as well as the DDQ score. The results showed that the LSAS score was positively correlated with the VAS score (*r* = 0.588, *p* < 0.001) and “Desire and Intention”(*r* = 0.282, *p* < 0.05). There was no correlation between the LSAS score and both “Negative reinforcement” (*r* = 0.042, *p* = 0.742), as well as “Control” score (*r* = −0.219, *p* = 0.084; Figure 3).

## Discussion

Participants under drug rehabilitation programs present with high number of drug-dependency complications compared with their counterparts. Previous studies report that SUD is significantly associated with SAD. Patients presenting with both SAD and SUD show severe complications compared with subjects with only a single disorder. Therefore, treatment of patients with SAD–AUD complications should not be limited to only a single disorder. Identification of possible similarities between the two treatments can provide a new therapeutic target for SAD–AUD patients. Correlation analysis based on LSAS, VAS, and DDQ scores shows a positive correlation between severity of SAD, VAS-craving, and “Desire and Intention,” indicating an interactive relationship between SAD and AUD. However, the results of the present study showed no correlation between “Negative reinforcement” and “Control” according to LSAS scores. This can be attributed to the small sample size used for the study or the high number of dropouts in this study. The findings showed significant differences in VAS-craving between the SAD group and the Non-SAD group. This implies that heroin addicts with SAD show high levels of fear during social interaction and social attachment. In addition, heroin addicts feel more inferior and nervous in social settings. Therefore, their inner loneliness increases, positive emotions decrease, and present with physiological arousal. These subjects thus rely on other ways to compensate for their inner positive emotions. Moreover, heroin addicts have the urge to compensate for their inner positive emotions when subjected to drug simulation cues. The pathological memory thus overlaps with physiological arousal and drug craving increases.

Studies report a positive association between OT and anxiety symptoms. For instance, a study comprising 29 patients with obsessive–compulsive disorder reported a higher OT level associated with more anxiety symptoms (34). Moreover, plasma OT level is positively correlated with anxiety composite scores in healthy women (35). On the contrary, Scantamburlo et al. (36) reported a negative correlation between anxiety symptoms and OT level in a group of 25 depressed patients. The findings of the present study exhibited a significantly high negative correlation between plasma OT levels and severity of SAD, which was consistent with the findings by Scantamburlo et al. (36). The severity of SAD and the baseline plasma OT levels were significantly different between SAD patients and Non-SAD patients. However, severity of SAD and the baseline plasma OT levels between the SAD control group and the SAD exercise group were not significantly different. This verified the correlation analysis results. Woolley et al. (21) conducted a study on the correlation between OT and heroin cue-induced cravings and reported that intranasal administration of a single OT dose to opioid users undergoing opioid replacement therapy was well-tolerated without significant effects on craving. This finding indicates a significant association between OT levels and heroin cues. Nikolaou et al. (37) reported a positive correlation between plasma OT levels of participants and scores based on COWS, VAS-Craving, and the Hamilton Anxiety scales. Contrary to these findings, Lin et al. (38) reported a negative correlation between plasma OT levels and craving levels in female heroin-dependent patients under methadone maintenance. The negative correlation was most significant in subjects with lower scores of novelty-seeking behaviors. VAS and DDQ scales were used in the present study to explore the relationship between cue-induced craving and plasma OT levels. The results showed that VAS-craving, “Desire and Intention,” and “Negative reinforcement” scores exhibited a negative relationship with plasma OT levels. Notably, VAS assessment results showed significant correlation with OT levels compared with the relationship between “Desire and Intention” and OT levels. VAS has fewer dimensions and short time; thus, it is easy to directly reflect a relationship. Moreover, the finding indicates that the DDQ scale is used to complement VAS in determining the degree of desire. A consistent negative correlation using both DDQ scale and VAS scale indicates the relationship between plasma OT levels and cue-induced cravings.

The results showed that aerobic combined with resistance exercise for a period of 8 weeks increased plasma OT levels of heroin withdrawal and alleviated SAD and cue-induced cravings. The efficacy of AE can be explained by the fact that aerobic exercise mimics the same bodily sensations elicited by anxiety reactions, such as increased heart rate, respiration, and perspiration (39). Therefore, exercise prompted the participants to differently evaluate their bodily responses (such as in a less threatening manner) compared with when they are not going through exercise training. In addition, repeated exposure to social stimuli at the gym could have caused habituation to social fear and changes in social cognitions, thus helping in alleviation of SAD symptoms. This indicates that exercising in an anxious situation results in alleviation of anxiety symptoms (39). Moreover, exercising (improving one's physical health) can induce decrease of negative judgments and enhance kindness toward oneself (self-compassion). A previous study reported that exercise can improve anxiety through repeated exposure, improved self-efficacy, and distraction (40). A randomized controlled trial conducted to improve SAD reported that first-time use of aerobic exercise and group lessons for 8 weeks effectively alleviated severity of SAD. Similarly, findings from a previous animal study showed that aerobic exercise increased OT levels in female mice, alleviated anxiety, and increased empathy. Aerobic exercise combined with resistance exercise is an effective alternative to improve physical health and reduce cravings of drug-dependent people. Furthermore, aerobics combined with resistance training can significantly alleviate anxiety and depression associated with drug dependency (41). Moreover, it can improve aerobic exercise performance, muscle strength, and endurance, and promote recovery from drug dependence (42).

The present study had several limitations. For instance, the sample size was too small, and most of the participants withdrew from the study. The study sought to recruit and screen more subjects. However, in order to control the rigor of the research during the screening process, a population of 1,026 people were screened based on the standard of “heroin only;” thus, only one-third of the subjects were eligible. This resulted in inclusion of a smaller number of subjects in the study. Furthermore, the effect of the female menstrual cycle was not explored despite its potential effect on plasma OT levels (43–45). Peripheral blood OT level was determined in this study and not the central nervous system level. Previous animal studies report that the peripheral and central nervous systems are coordinated. Furthermore, plasma osmolality was not evaluated, which may affect plasma OT levels. DDQ and VAS scales were used to explore the craving degree to ensure the findings were accurate. However, this study lacks objectivity, owing to lack of objective indicators for strong demonstration. Although the preliminary experiments explored the clues that induce changes in blood pressure and heart rate during craving, these findings are not included in the study.

Although the relationship between OT level and SAD and heroin addiction has been extensively studied, only a few studies have explored the relationship between endogenous OT and the interaction between SAD and heroin addiction. Therefore, this is the first study to explore the effect of exercise in heroin addicts with SAD. The findings of this study indicate that adjuvant therapy, such as exercise, can improve the level of endogenous OT in drug withdrawal patients with SAD.

## Conclusion

Plasma OT is a potential peripheral biomarker for prediction of severity of social anxiety in female heroin withdrawal Patients. Moreover, exercise can be used as an adjuvant therapy to increase the level of OT in heroin withdrawal patients and alleviate SAD and cue-induced craving.

## Data Availability Statement

The raw data supporting the conclusions of this article will be made available by the authors, without undue reservation.

## Ethics Statement

The studies involving human participants were reviewed and approved by the Institutional Review Committee of Hunan Normal University approved. The patients/participants provided their written informed consent to participate in this study.

## Author Contributions

JW, JZ, LZ, and YG conceived and designed the experiments. JT, TH, and JL screened experimental subjects, signed the informed consent process, and conducted the exercise intervention. All authors contributed to the article and approved the submitted version.

## Funding

The authors declare that this study received funding from the National Key Research and Development Program of China (Grant No. 2016YFC0800908), the Research and Innovation Program for Postgraduates of Physical Education College of Hunan Normal University (TYCX2019B007), the Scientific Research Project of Hunan Provincial Department of Education (19C1133), the Philosophy and Social Science Fund Project of Hunan Province (18YBQ088), and the China Postdoctoral Science Foundation Funded Project (2017M622580). The funder was not involved in the study design, collection, analysis, interpretation of data, the writing of this article, or the decision to submit it for publication.

## Conflict of Interest

The authors declare that the research was conducted in the absence of any commercial or financial relationships that could be construed as a potential conflict of interest.

## Publisher's Note

All claims expressed in this article are solely those of the authors and do not necessarily represent those of their affiliated organizations, or those of the publisher, the editors and the reviewers. Any product that may be evaluated in this article, or claim that may be made by its manufacturer, is not guaranteed or endorsed by the publisher.
